# Catalase improves saccharification of lignocellulose by reducing lytic polysaccharide monooxygenase-associated enzyme inactivation

**DOI:** 10.1007/s10529-015-1989-8

**Published:** 2015-11-05

**Authors:** Brian R. Scott, Hong Zhi Huang, Jesper Frickman, Rune Halvorsen, Katja S. Johansen

**Affiliations:** Biomass Enzyme Discovery, Novozymes Inc., 1445 Drew Ave, Davis, CA 95618 USA; Bioenergy Asia, Novozymes (China) Investment Co. Ltd., 14 Xinxi Road, Shangdi Zone, Haidian District, Beijing, 100085 China; Biomass Application Discovery, Novozymes North America, 77 Perry’s Chapel Church Road, Franklinton, NC 27525 USA; Biofuels Technology, Novozymes A/S, Krogshøjvej 36, 2880 Bagsværd, Denmark; Division of Industrial Biotechnology, Chalmers University of Technology, Kemivägen 10, 412 96 Göteborg, Sweden

**Keywords:** Catalase, Cellulase, Fenton chemistry, Kinetic modelling, Lytic polysaccharide monooxygenase, Pretreated wheat straw, Reactive oxygen species

## Abstract

**Objectives:**

Efficient enzymatic saccharification of plant cell wall material is key to industrial processing of agricultural and forestry waste such as straw and wood chips into fuels and chemicals.

**Results:**

Saccharification assays were performed on steam-pretreated wheat straw under ambient and O_2_-deprived environments and in the absence and presence of a lytic polysaccharide monooxygenase (LPMO) and catalase. A kinetic model was used to calculate catalytic rate and first-order inactivation rate constants of the cellulases from reaction progress curves. The addition of a LPMO significantly (*P* < 0.01, Student’s *T* test) enhanced the rate of glucose release from 2.8 to 6.9 h^−1^ under ambient O_2_ conditions. However, this also significantly (*P* < 0.01, Student’s *T* test) increased the rate of inactivation of the enzyme mixture, thereby reducing the performance half-life from 65 to 35 h. Decreasing O_2_ levels or, strikingly, the addition of catalase significantly reduced (*P* < 0.01, Student’s *T* test) enzyme inactivation and, as a consequence, higher efficiency of the cellulolytic enzyme cocktail was achieved.

**Conclusion:**

Oxidative inactivation of commercial cellulase mixtures is a significant factor influencing the overall saccharification efficiency and the addition of catalase can be used to protect these mixtures from inactivation.

**Electronic supplementary material:**

The online version of this article (doi:10.1007/s10529-015-1989-8) contains supplementary material, which is available to authorized users.

## Introduction

Cellulose is the planet’s most abundant biopolymer and is found in plant cell walls in a strong network with other polysaccharides and the aromatic polymer lignin. Overcoming the recalcitrance of the plant cell wall (and of cellulose in particular) to depolymerisation remains a target for optimisation and effective use of lignocellulosic biomass as an industrial feedstock. As several full-scale factories producing lignocellulosic ethanol have come online recently around the world, the importance of guiding the industry towards optimal process conditions cannot be overestimated.

Various factors that negatively impact the hydrolysis efficiency of cellulase enzymes on pretreated plant cell wall material have been reported (reviewed by Yang et al. 2001). These include substrate-related factors, such as the presence of hemicellulose, cellulose crystallinity, degree of polymerization and increasing recalcitrance as a function of cellulose conversion, and enzyme-related factors, including product inhibition, inhibition from pretreatment-derived xylooligomers and phenols, inactivation and non-productive binding. However, the relative importance of these factors is insufficiently understood due in part to the variety of substrates, enzymes and assay conditions employed in these studies.

Lytic polysaccharide monooxygenases (LPMOs), such as AA9, carry out oxidative cleavage of glycosidic bonds and boost the activity of glycosyl hydrolases. However, in the absence of cellulose and in the presence of O_2_ and a reducing agent, AA9 will release superoxide as a product of each redox cycle (Kjaergaard et al. 2014). Superoxide is spontaneously transformed into H_2_O_2_ that reacts with transition metals such as iron and copper to form highly toxic hydroxyl radicals and other reactive oxygen species. Catalase (EC 1.11.1.6) fulfils a crucial role in biology as it terminates chains of radical chemistry by the dismutation of H_2_O_2_ into water and O_2_.

The importance of LPMO and O_2_-associated inactivation of cellulase mixtures is shown here for the first time, in addition to the ability of catalase to mitigate this process.

## Materials and methods

Unless otherwise stated, all reagents were laboratory grade. Steam-pretreated wheat straw was purchased at Lund University (kindly prepared by Mats Galbe). All commercial enzymes were obtained from Novozymes A/S. The aldose oxidase used in this study was cloned from *Microdochium nivale* (Genbank Accession BD103535) and expressed from *Fusarium venenatum* (Xu et al. 2001). Cloning and expression of the *Thermoascus aurantiacus* (Ta) AA9 (Accession ABW56451) was performed as previously described (Dotson et al. 2007). Catalase from *Thermoascus aurantiacus* (Accession DD046677) was expressed in *Aspergillus niger* and purified as described previously (Haruhiko and Sadaji 2004). The β-glucosidase from *Aspergillus fumigatus* (Af) (Accession EAL88289) was cloned and expressed from *Aspergillus oryzae* as described elsewhere (Teter et al. 2008).

### Enzymatic saccharification of lignocellulose under low O_2_ conditions

A disposable 280 L Atmosbag (Sigma Aldrich) was used for conducting saccharification experiments at 20 g scale under controlled O_2_ levels. First, the level of dissolved O_2_ in the reactions could be decreased to 1 % relative to the concentration in ambient air (as measured by Mettler Toledo O_2_ electrode) by flushing the tent with N_2_ and degassing all liquids prior to the experiment. These conditions caused control reactions with cellobiose and aldose oxidase to be significantly reduced in a 1 h experiment (Supplementary Fig. 1). Duplicate samples of steam pretreated wheat straw (Lund University) were hydrolysed at 10 % (w/v) dry matter content (3.6 % initial cellulose) at around pH 5 at 50 °C using free-fall mixing (Boekel Scientific, Big Shot III Hybridization oven model 230402) for up to 144 h.

Four doses of Cellic CTec3, 2.8, 5.6, 8.4 and 11.2 mg protein/g cellulose, were tested with and without addition of a constant dose of 0.22 mg Ta catalase/g cellulose. Half of the samples set were tested under standard conditions (without control of O_2_ levels) and the other half was tested under a N_2_ saturated atmosphere as described above. The pH was measured and adjusted daily and the glucose concentrations in samples withdrawn from the test tubes were determined. pH was adjusted using 1 M KOH and 75 mg Lactrol per kg slurry was added to prevent contamination by lactic acid bacteria.

The effect of LPMO activity was determined in a similar experimental set-up with enzyme blends consisting of 80 % Celluclast 1.5L, 10 % *A. fumigatus* β-glucosidase, and 10 % *Thermoascus aurantiacus* AA9 or BSA (96 % Sigma) by protein mass. The total protein dose in each experiment was 8.4 mg protein/g cellulose.

For the experiment with added H_2_O_2_, lids for the reaction tubes with a septum were used. A defined amount of H_2_O_2_ was added to the reaction mixture using Hamilton syringes after about 18 h of incubation at which time the reaction mixtures were liquefied. H_2_O_2_ was added four times at intervals of about 3.5 h during which the tubes were held at 50 °C.

### Determination of free sugar

The concentration of glucose was determined by HPLC analysis using an Aminex HPX 87H column, 5 mM H_2_SO_4_ isocratic eluent and refractive index detection. Fractional cellulose conversion to glucose concentrations were calculated by dividing the glucose concentrations measured by HPLC at each time point by the initial concentration of glucose equivalents in the substrate.

### Kinetic modelling

**Fig. 1:**
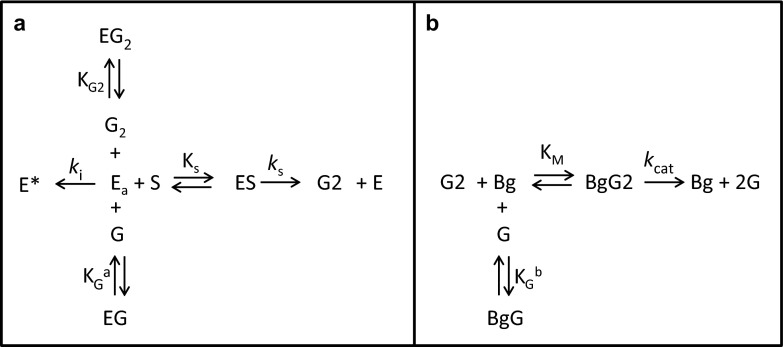
*Arrow* diagrams describing the two-stage Michaelis–Menten kinetic model with competitive inhibition and cellulase inactivation. A model describing the conversion of cellulose (S) to cellobiose (G2) catalyzed by active cellulase enzyme (E_a_) is shown in *panel a*. The cellulase in this model is subject to competitive inhibition by both glucose (G) and cellobiose (G2) and time-dependent inactivation to E*. The model process by which cellobiose is converted to glucose (G) by β-glucosidase (Bg) is shown in *panel b*. The β-glucosidase is subject to competitive inhibition by glucose

A two-stage kinetic model (Fig. 1) was used to analyze cellulose hydrolysis progress curves generated under ambient and reduced O_2_ conditions. The first stage in the model involves the conversion of cellulose to cellobiose (panel a). As described, all of the components in the commercial cellulases tested here, except for β-glucosidase(s), contributed to the formation of cellobiose. Similarly, only β-glucosidase, which accounted for either 5 or 10 % of the protein mass of the commercial enzyme preparations tested here, catalyzed the conversion of cellobiose to glucose (panel b). Both stages were assumed to follow Michaelis–Menten kinetics. It was also assumed that the cellulases in Stage 1 were subject to competitive inhibition by glucose (K_G_^a^) and cellobiose (K_G2_) and subject to first-order inactivation (*k*_i_) to the inactive form, E*. β-glucosidase, that was subject to competitive inhibition by glucose (K_G_^b^).

The mechanism of cellulose hydrolysis has been described using Michealis-Menten kinetics with product inhibition by others (reviewed by Sousa et al. 2011). This model was employed because it provided a suitable framework for interpreting the progress curves generated here under a limited range of substrate concentrations and enzyme doses. Additional complexities, such as enzyme adsorption onto cellulose (Scheiding et al. 1984, Philippidis et al. 1992, Nidetzky and Steiner 1993) and increasing recalcitrance of the substrate (Drissen et al. 2007) reported by others were not included in this model but are discussed below. The rate equations for the first (Eq. 1) and second (Eq. 2) stages of the model are shown below.

1 $$ - \frac{\text{dS}}{\text{dt}} = \frac{{{\text{k}}_{\text{s}} \cdot {\text{E}}_{\text{a}} \cdot {\text{S}}}}{{{\text{S}} + {\text{K}}_{\text{s}} \left( {1 + \frac{\text{G}}{{{\text{K}}_{\text{G}}^{\text{a}} }} + \frac{{{\text{G}}2}}{{{\text{K}}_{{{\text{G}}2}} }}} \right)}} = \frac{{{\text{dG}}2}}{\text{dt}} \times \frac{{342   {\text{g}}/{\text{mol}}}}{{324   {\text{g}}/{\text{mol}}}} $$

where, S is the substrate cellulose (g/l); t is time (h); *k*_s_ is the cellulase catalytic rate constant (h^−1^); E_a_ is the concentration of active cellulase (g/l); K_s_ is the cellulase Michaelis–Menten constant (g/l); G is glucose (g/l); K_G_^a^ is the competitive glucose inhibition constant for cellulase (g/l); G2 is cellobiose (g/l); K_G2_ is the competitive cellobiose inhibition constant for cellulase (g/l)

2 $$ \frac{\text{dG}}{\text{dt}} = \frac{{k_{\text{cat}}   {\text{Bg  G}}2}}{{G2 + {\text{K}}_{\text{M}} \left( {1 + \frac{\text{G}}{{{\text{K}}_{\text{G}}^{\text{b}} }}} \right)}} \times \frac{{360   {\text{g}}/{\text{mol}}}}{{342   {\text{g}}/{\text{mol}}}} $$

where, *k*_cat_ is the β-glucosidase catalytic rate constant (h^−1^); Bg is the concentration of β-glucosidase (g/l); K_M_ is the β-glucosidase Michaelis–Menten constant (g/l); K_G_^b^ is the competitive glucose inhibition constant for β-glucosidase (g/l)

Inactivation of cellulases under the assay conditions described above was assumed to be first-order and is described in Eq. 3. A cellulase performance half-life (t_1/2_) was calculated according to Eq. 4. β-glucosidase activity was assumed to be stable.

3 $$ \frac{{{\text{dE}}_{\text{a}} }}{\text{dt}} = - k_{\text{i}} \cdot {\text{E}}_{\text{a}} $$

where, *k*_i_ is the cellulase inactivation rate constant (h^−1^)

4 $$ {\text{t}}_{1/2} = - \frac{0.693}{{k_{\text{i}} }} $$

The differential equations were applied to each data set using a 4th order Runge–Kutta numerical integration using Microsoft Excel. The model was used to fit all doses of enzyme tested under a given set of experimental conditions by varying, *k*_s_ and *k*_i_. Optimal values of *k*_s_ and *k*_i_ were determined simultaneously using the Excel Solver by minimization of least squres. Other parameters in the kinetic model were fixed to the values shown in Table 1.

**Table 1:** Values for individual kinetic parameters that were fixed in all model fits

Parameter	Value
K_s_	42 g/l
K_G_^a^	13 g/l
K_G2_	3 g/l
*k*_cat_	100 h^−1^
K_M_	2 g/l
K_G_^b^	1 g/l

## Results and discussion

### The effect of O_2_ deprivation

**Fig. 2:**
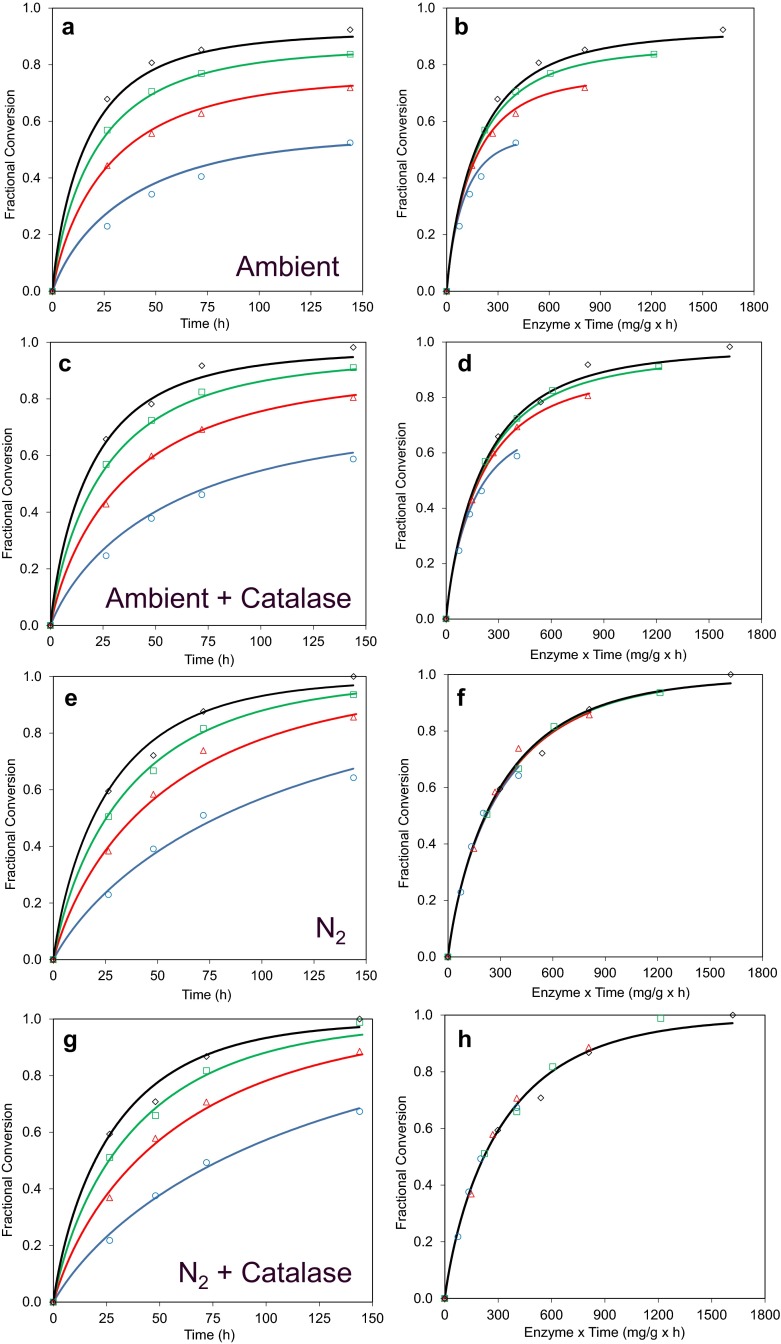
Effects of O_2_ limitation and catalase addition on CTec3 cellulose hydrolysis progress curves. Pretreated wheat straw was incubated with 2.8 (*blue circles*), 5.6 (*red triangles*), 8.4 (*green squares*) and 11.2 mg protein/g cellulose (*black diamonds*) of CTec3 for 144 h at 50 °C, pH 5. Reactions were carried out in ambient air (*panels a* and *c*) and O_2_-deprived (*panels e* and *g*) conditions. Similarly, the effects of adding catalase under each of these conditions are shown in *panels c* and *g*, respectively. To better illustrate time-dependent enzyme inactivation under each of these conditions, the conversion data are plotted as a function of enzyme dose x time in *panels b, d, f* and *h*. Model fits to these data are shown by lines colour-matched to the data

Steam-pretreated wheat straw was saccharified using the commercial enzyme product Cellic CTec3 at four different dosages and the fractional conversion of cellulose to glucose plotted as a function of time (Fig. 2). Progress curves of the data obtained for the ambient assay condition are shown in Fig. 2a. The initial rate of glucose release was, as expected, positively correlated with the enzyme dosage used and approximately 90 % cellulose conversion to glucose was observed by 144 h using the highest enzyme dose. It also appeared that the glucose release data over time resulting from each enzyme dose in Fig. 2a approached different asymptotic values, indicating that substrate depletion alone did not account for the decrease in glucose release over time. When the conversion data were plotted as a function of enzyme dose multiplied by time (Et) as shown in Fig. 2b, it was clear that the conversion data did not superimpose but rather deviated from a common Et relationship. This observation is consistent with time-dependent inactivation of the enzyme (Selwyn 1965) and kinetic modelling of these data indicated that the cellulase half-life was 39 h under these conditions (Table 2). When CTec3 was tested under limited O_2_ conditions (Fig. 2, panels e, f), the cellulase half-life increased significantly (*P* < 0.01, Student’s *T* test) to the extent that no significant level of enzyme inactivation was observed. Interestingly, the cellulase catalytic rate constant measured under O_2_-limited conditions (11.0 h^−1^) was significantly (*P* < 0.01, Student’s *T* test) lower than under ambient conditions (16.5 h^−1^), indicating that the reduced O_2_ levels also resulted in slower cellulase catalysis.

**Table 2:** Parameter values from model fits to CTec3 progress curves shown in Fig. 2

Air	Catalase	*k*_s_ (h^−1^)	*k*_i_ (× 10^−3^ h^−1^)	t_1/2_ (h)
Ambient	No catalase	16.5 ± 1.5	17.7 ± 2.7	39 ± 6
Ambient	Catalase	14.9 ± 1.2	9.3 ± 1.8^a^	75 ± 15^b^
N_2_	No catalase	11 ± 0.9^a^	1.2 ± 2.1^a^	582 ± 981^a^
N_2_	Catalase	10.5 ± 0.6^a^	0 ± 1.3^a^	n/a^a^

The model was fitted to each progress curves shown in Fig. 2 by varying *k*_s_ and *k*_i_. Values shown are best fit values and their associated standard deviations. Statistically significant differences relative to the ambient air and no catalase control, as determined using Student’s *T* test, are indicated

^a^ *P* value < 0.01 relative to value of equivalent parameter measured under ambient air conditions without catalase

^b^ *P* value < 0.05 relative to value of equivalent parameter measured under ambient air conditions without catalase

These results indicate that oxidative reactions harmful to the cellulolytic enzyme cocktail take place in the reaction mixture. Cellic CTec3 contains significant amounts of AA9 which requires O_2_ for activity and previous reports have pointed to the need for O_2_ to be available for optimal saccharification efficiency (Cannella and Jorgensen 2014, Müller et al. 2015). In line with these reports, O_2_ deprivation (Fig. 2e, f) significantly reduced the catalytic rate constant. However, a marked increase in enzyme half-life was also observed. The combined effect is that reactions conducted in ambient conditions saccharified faster; however, given sufficient incubation time, the highest glucose release was observed in the low O_2_ regime (Supplementary Fig. 2).

The rate of glucose production decreases markedly as cellulose hydrolysis proceeds and that this cannot be accounted for simply by substrate depletion. Product inhibition, substrate transformation, non-productive binding and enzyme inactivation have been proposed to account for this phenomenon. Importantly, the time-dependent enzyme inactivation observed above is distinct from conversion-dependent phenomena, which follow a common Et relationship. While such effects were not addressed in our kinetic model, any conversion-dependent phenomena, such as increasing substrate recalcitrance (Desai and Converse 1997), Drissen et al. 2007), would be captured in the cellulase product inhibition constant (K_G_^a^), which was fixed for modelling purposes, along with true glucose inhibition effects.

A Langmuir-like dependence of glucose production rate as a function of cellulase dose has been reported by others (Philippidis et al. 1993), Drissen et al. 2007). However, the observation here that progress curves measured under O_2_-limited conditions follow a common Et relationship indicates that the doses used did not reach the maximum binding capacity of the substrate. If this were the case, then a deviation from the Et relationship in which higher doses fall below the trend established by lower doses would be observed. In all likelihood, if even higher cellulase doses were tested, such a deviation in the Et relationship would be observed at some point. However, the absence of such an observation here supports the omission of a Langmuir-type dependency on cellulase catalytic rate from this model.

### The effect of catalase addition

Catalase function is essential to control oxidative stress in living systems, where H_2_O_2_ is a by-product of mitochondrial electron transport, β-oxidation of fatty acids and photorespiration (Kocabas et al. 2008). In addition to the biotic (enzymatic) generation of H_2_O_2_, this compound is also an unavoidable reactive oxygen species produced by abiotic (non-enzymatic) routes during aerobic degradation of organic matter (Remucal and Sedlak 2011). H_2_O_2_is either directly or indirectly harmful to enzymes and inhibits the activity of cellulases (Reese and Mandels 1980). The effect of adding the thermostable catalase from *Thermoascus aurantiacus* was therefore tested.

When catalase was added (Fig. 2 c and d) under ambient air conditions, the deviation in the Et relationship decreased substantially. Modelling of these data indicated that the addition of catalase significantly (*P* < 0.05, Student’s *T* test) increased the enzyme half-life to 75 h. No significant effect on the cellulase catalytic rate constant was observed, implying that catalase had no net effect on the rate of cellulase catalysis under these conditions. Addition of catalase under O_2_-deprived conditions (panel g and h) had no detectable effects.

### LPMO activity is important for inactivation

In order to probe the impact of LPMO activity on the O_2_- and catalase-related inactivation observed above, enzymes mixtures with and without supplemental AA9 were tested. Celluclast, which is a wild-type *Trichoderma reesei* whole enzyme mixture, was used as an LPMO-poor cellulase cocktail. Mixtures were prepared containing 80 % Celluclast by protein mass and 10 % β-glucosidase to ensure rapid and complete conversion of cellobiose to glucose, thereby avoiding product inhibition of the cellobiohydrolases and simplifying product quantification. The remaining 10 % of the mix comprised either the Ta AA9 or an inert protein in the form of bovine serum albumin.

**Fig. 3:**
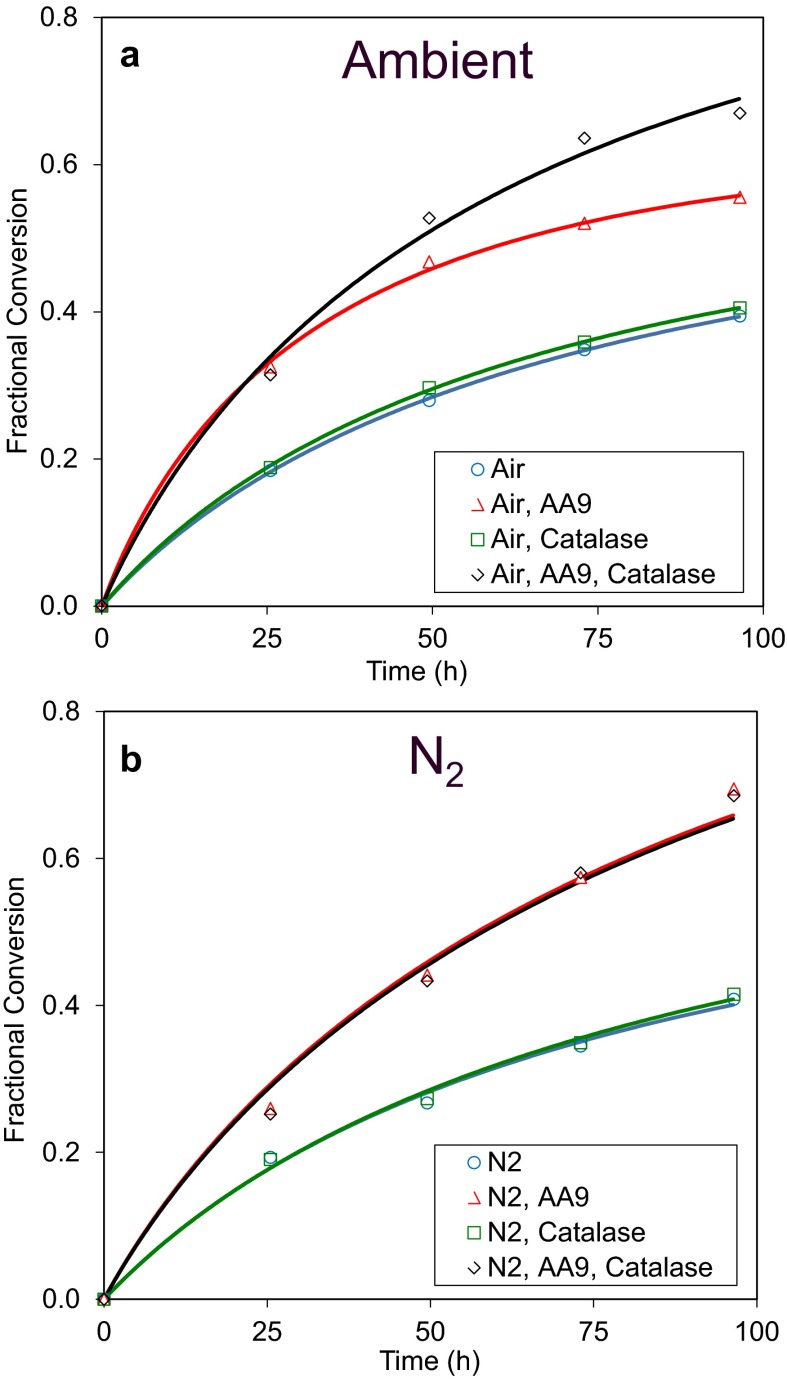
Effect of catalase on cellulose hydrolysis performance in presence and absence of AA9 and limited O_2_. Pretreated wheat straw was incubated with 8.4 mg total enzyme protein/g cellulose at 50 °C, pH 5 for 96 h under conditions of ambient air (*panel a*) or after purging with N_2_ (*panel b*). The enzyme cocktail consisted of 80 % Celluclast 1.5L, 10 % β-glucosidase, and 10 % either LPMO (Ta AA9) or 10 % BSA. In the indicated samples, Ta catalase was added at a concentration of 0.22 mg enzyme/g cellulose. Model fits to these data are shown by lines colour-matched to the data

The progress curves for these experiments are shown in Fig. 3 and values for the kinetic parameters shown in Table 3. The *k*_s_ and t_1/2_ of the Celluclast + β-glucosidase were 2.8 h^−1^ and 65 h, respectively, under ambient air (panel a). Addition of the AA9 increased the catalytic rate to 6.9 h^−1^ and resulted in an enzyme mixture with a lower t_1/2_ (35 h), showing that the AA9 is closely associated with both the rate of catalysis and the rate of inactivation of the cellulase mixture. Inactivation of the cellulase mixture containing AA9 was reduced significantly (*P* value <0.01, Student’s *T* test) when catalase was added while catalase addition, as before, had no significant effect on *k*_s_. Similarly, catalase addition had no effect when added simply to a blend of Celluclast +β-glucosidase, indicating that catalase increases the stability of a cellulase mixture only when an AA9 is present.

**Table 3:** Parameter values from model fits to cellulose hydrolysis progress curves shown in Fig. 3

Air	AA9	Catalase	*k*_s_ (h^−1^)	*k*_i_ (× 10^−3^ h^−1^)	t_1/2_ (h)
Ambient	–	–	2.8 ± 0.1	9.6 ± 0.8	65 ± 6
Ambient	+	–	6.9 ± 0.4^a^	17.8 ± 2.0^a^	35 ± 4^a^
Ambient	–	+	2.9 ± 0.1	9.9 ± 0.7	63 ± 5
Ambient	+	+	7.2 ± 1.0^a^	4 ± 3.8^b^	154 ± 160^b^
N_2_	–	–	2.9 ± 0.3	7.9 ± 3.4	78 ± 37
N_2_	+	–	4.6 ± 0.6^a,c^	0 ± 3.3^a^	n/a^a^
N_2_	–	+	2.6 ± 0.2	6.9 ± 2.6	90 ± 37
N_2_	+	+	4.6 ± 0.4^a,c^	0 ± 2.4^a^	n/a^a^

The model was fitted to each progress curves shown in Fig. 3 by varying *k*_s_ and *k*_i_. Values shown are best fit values and their associated standard deviations. Statistically significant differences, as determined using Student’s *T* test, are indicated

^a^ *P* value < 0.01 relative to value of equivalent parameter measured under ambient air conditions without catalase

^b^ *P* value < 0.01 relative to value of equivalent parameter measured under ambient air conditions, with AA9 and without catalase

^c^ *P* value < 0.01 relative to value of equivalent parameter measured under O_2_-deprived conditions without AA9 or catalase

Similar experiments done under O_2_-deprived conditions are shown in panel b. The *k*_s_ and t_1/2_ of the mixture without Ta AA9 and catalase under O_2_-deprived conditions (2.8 h^−1^ and 78 h, respectively) were similar to those measured under ambient conditions. Addition of AA9 under O_2_-limiting conditions increased the catalytic rate constant of the enzyme mixture from 2.8 to 4.6 h^−1^. However, this *k*_s_ was lower than that measured under ambient conditions (6.9 h^−1^) indicating that the reduced O_2_ levels limited the catalytic activity of the AA9. No significant changes in t_1/2_ were observed upon addition of AA9 or catalase under O_2_-limited conditions. This further demonstrates that the effects of O_2_ here, both on the catalytic rate and inactivation of cellulase mixtures, are AA9-dependent.

### Addition of H_2_O_2_ hampers cellulolytic activity

**Fig. 4:**
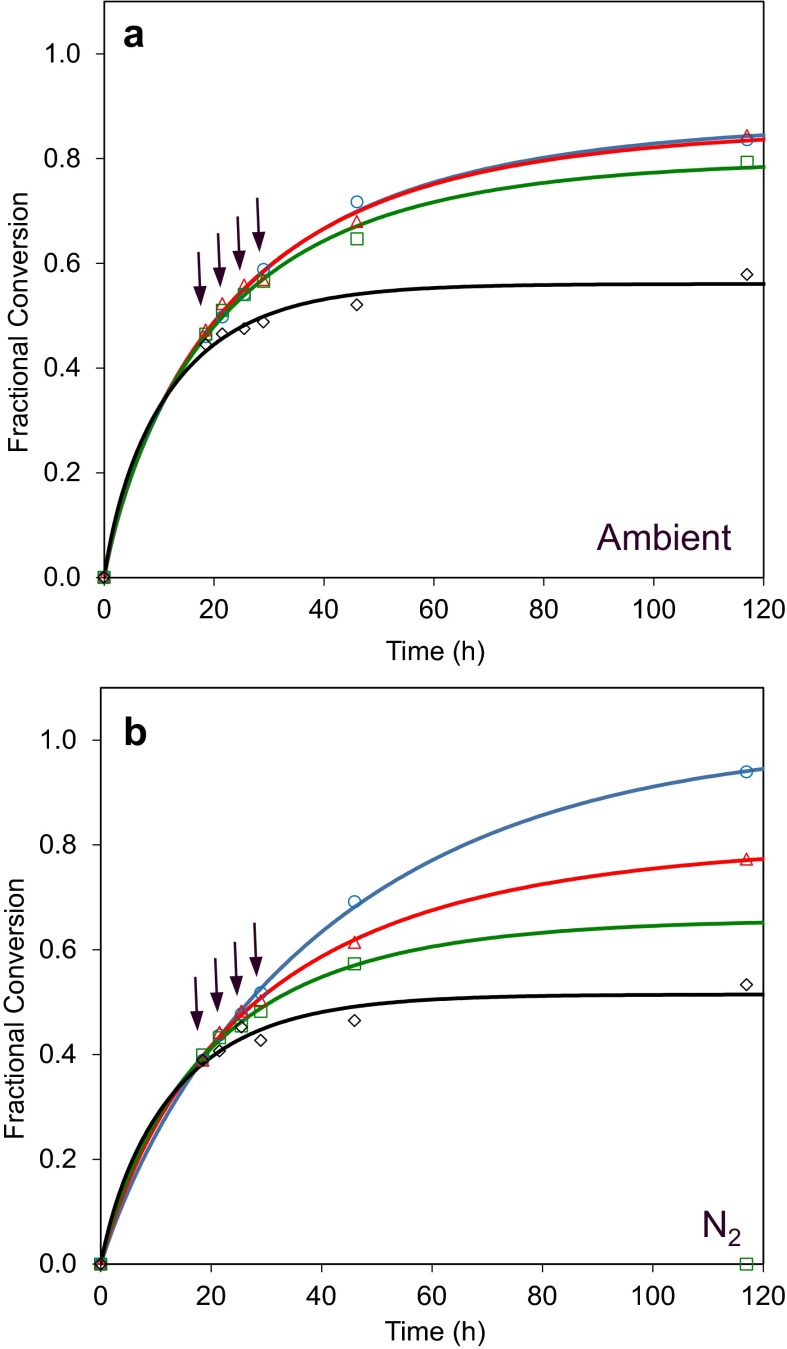
Effects of exogenous H_2_O_2 _addition on cellulase performance under ambient air and limited O_2_ conditions. CTec3 was incubated with pretreated wheat straw in the presence of 0 (blue circles), 0.01 (*red triangles*), 0.1 (*green squares*) and 1 (*black diamonds*) mg H_2_O_2_/g slurry added four times (as indicated by the *arrows*) after the cellulosic material was liquefied. Effects of H_2_O_2_ were tested under ambient air (*panel a*) and after purging with N_2_ (*panel b*). For this experiment lids for the reaction tubes with a septum were used and the indicated amount of H_2_O_2_ was added to the reaction mixture using Hamilton syringes. The first addition was made after about 18 h of incubation at which time the reaction mixtures were liquefied. H_2_O_2_ was added four times at intervals of about 3.5 h during which the tubes continued incubation at 50 °C

The addition *ofT. aurantiacus* catalase in the experiments above clearly reduced inactivation of a cellulase mixture containing AA9 under ambient O_2_ conditions. Yet catalase addition had no apparent effect on catalytic rate, implying catalase interrupts O_2_-dependent radical reactions mediated by the AA9 that are damaging to one or more components of the enzyme mixture but that are distinct from the AA9 catalytic activity on cellulose. To better understand the effects H_2_O_2_may have in this system, CTec3 was incubated with pretreated wheat straw under both ambient and O_2_-deprived conditions with and without different doses of H_2_O_2_. These progress curves are shown in Fig. 4.

The lowest dose, 0.01 mg H_2_O_2_/g slurry, did not have any impact on glucose release in the ambient condition [Fig. 4a, blue (control) and red markers). In contrast, this dose of H_2_O_2_ had a strong negative effect under the N_2_ condition as it led to a reduction in conversion of approximately 10 % (Fig. 4c). At 0.01 mg H_2_O_2_/g slurry, the amount of H_2_O_2_ added at each injection is only 1/10 of the concentration previously found to reduce the activity of *Trichoderma* cellulases by 50 % in assays using Avicel as the substrate (Reese and Mandels 1980). Still, the final yield of glucose is severely reduced to a level below the condition with ambient air (Supplementary Fig. 2). The addition of catalase fully prevented the loss of cellulolytic activity (Supplementary Fig. 3).

The robustness of the system was tested by adding higher dosages of H_2_O_2_ with or without catalase present (Fig. 4). In the absence of catalase, the addition of two doses of 1 mg H_2_O_2_/g had a catastrophic effect on glucose release regardless of the level of O_2_ tested here (Fig. 4 a, b, black lines). The data show that the fixed concentration of catalase used in this study can detoxify both the 0.01 and the 0.1 mg added H_2_O_2_/g whereas the highest concentration 1 mg/g is too high for efficient detoxification in the O_2-_deprived reactions (Supplementary Fig. 3).

The formation reaction paths of H_2_O_2_ are dependent on physical parameters such as temperature, dry matter content and on chemical parameters such as pH, concentration of transition metal and importantly the availability of O_2_. H_2_O_2_ will spontaneously react with a large number of chemical species in the reaction mixture and the absolute concentration may therefore be relatively low compared to the flux. Indeed, it is highly likely that it is the hydroxyl radical derived from H_2_O_2_ rather than H_2_O_2_ itself that leads to oxidative inactivation of enzymes. A transient concentration in the range of 0.01 mg/g of slurry under O_2_ deprivation seems to have a larger impact than what is seen in ambient air but more sophisticated analyses are required to determine the O_2_-dependent flux in H_2_O_2_.

**In conclusion,** oxidative inactivation of commercial cellulase mixtures is a significant factor influencing the overall saccharification efficiency and the addition of catalase can successfully be used to protect cellulases mixtures from inactivation.

## Electronic supplementary material

Supplementary material 1 (DOCX 197 kb)
