# Supplementary material for: Catalase improves saccharification of lignocellulose by reducing lytic polysaccharide monooxygenase-associated enzyme inactivation
Source: Biotechnol Lett. 2015 Nov 5;38:425–34. doi: 10.1007/s10529-015-1989-8 (PMC4767857; doi:10.1007/s10529-015-1989-8)
Supplement: Supplementary file 1 — Supplementary material 1 (DOCX 197 kb) [file 10529_2015_1989_MOESM1_ESM.docx]

Supplementary Data

**Supplementary Fig.1** The reduction in cellobiose concentration through oxidation by aldose oxidase either in ambient air or under ~1% O_2_ tension relative to ambient air. An inflatable 280 L Atmosbags for experimental work in a controlled environment was used for assembling reaction mixtures. All liquids were vacuum degassed before they were placed in the nitrogen-filled bag and then flushed with N_2_. Triplicate samples of a solution of 1 g cellobiose/L was incubated with 0.1 g *Microdochium nivale* aldose oxidase L at 50°C for 1h and then heat inactivated for 10 min at 90°C. The concentration of remaining cellobiose was determined on a Dionex ICS 5000, 345-2050 equipped with Dionex CarboPac PA1 (4x50mm guard and 4x250mm analytical).

**Supplementary Fig. 2** Effects of O_2_ limitation on CTec3 saccharification of steam pretreated wheat straw. Duplicate samples of steam pre-treated wheat straw (Lund University) were hydrolysed at 10% dry matter content at around pH 5 at 50°C using free-fall mixing (Boekel Scientific, Big Shot III Hybridization oven model 230402) for up to 144h. A constant dose of CTec3 of 8.4 mg protein/g cellulose was used. Samples were run under standard conditions (with no control of O_2_ levels) or under a nitrogen saturated atmosphere as described above. The pH was measured and adjusted daily and the glucose and xylose concentrations in samples withdrawn from the test tubes were determined. pH was adjusted using 1 M KOH and 75 mg Lactrol per kg slurry was added to prevent contamination by lactic acid bacteria.

**Supplementary Fig. 3** Effects of exogenous H_2_O_2_addition on cellulase performance under ambient air and limited O_2_ conditions in the presence of catalase. CTec3 was incubated with pretreated wheat straw in the presence of 0 (blue circles), 0.01 (red triangles), 0.1 (green squares) and 1.0 (black diamonds) mg H_2_O_2_/g slurry added four times (as indicated by the arrows) after the cellulosic material was liquefied. Effects of H_2_O_2_ were tested under ambient air (panel a) and after purging with N_2_ (panel b). Catalase was added along with CTec3 at a dose of 0.22 mg protein/cellulose to all reactions shown in panels a and b.
